# Activation of the Hedgehog signaling pathway leads to fibrosis in aortic valves

**DOI:** 10.1186/s13578-023-00980-1

**Published:** 2023-03-02

**Authors:** Dongsheng Gu, Arvin H. Soepriatna, Wenjun Zhang, Jun Li, Jenny Zhao, Xiaoli Zhang, Xianhong Shu, Yongshi Wang, Benjamin J. Landis, Craig J. Goergen, Jingwu Xie

**Affiliations:** 1 Department of Pediatrics, Indiana University School of Medicine, Wells Center for Pediatric Research, 1040 W. Walnut Street., Indianapolis, IN 46202 USA; 2 Purdue University Weldon School of Biomedical Engineering, 206 S. Martin Jischke Drive, Room 3025, West Lafayette, IN 47907 USA; 3 School of Engineering, Center for Biomedical Engineering, Brown University, 184 Hope Street, Providence, RI 02912 USA; 4 Shanghai Institute of Cardiovascular Diseases, Zhongshan Hospital Fudan University, 180 Fenglin Road, Shanghai, 200032 China; 5 Boston University School of Medicine, 72 E. Concord St., Boston, MA 02118 USA; 6 Present Address: Basic and Translational Cancer Review Branch (BTC), Division of Basic and Integrative Biological Sciences (DBIB), Center for Scientific Review, National Institutes of Health, 6701 Rockledge Drive, Bethesda, MD 20892 USA

**Keywords:** Aortic valve, Smoothened, Hedgehog, Fibrosis, Stenosis

## Abstract

**Background:**

Fibrosis is a pathological wound healing process characterized by excessive extracellular matrix deposition, which interferes with normal organ function and contributes to ~ 45% of human mortality. Fibrosis develops in response to chronic injury in nearly all organs, but the a cascade of events leading to fibrosis remains unclear. While hedgehog (Hh) signaling activation has been associated with fibrosis in the lung, kidney, and skin, it is unknown whether hedgehog signaling activation is the cause or the consequence of fibrosis. We hypothesize that activation of hedgehog signaling is sufficient to drive fibrosis in mouse models.

**Results:**

In this study, we provide direct evidence to show that activation of Hh signaling via expression of activated smoothened, SmoM2, is sufficient to induce fibrosis in the vasculature and aortic valves. We showed that activated SmoM2 -induced fibrosis is associated with abnormal function of aortic valves and heart. The relevance of this mouse model to human health is reflected in our findings that elevated GLI expression is detected in 6 out of 11 aortic valves from patients with fibrotic aortic valves.

**Conclusions:**

Our data show that activating hedgehog signaling is sufficient to drive fibrosis in mice, and this mouse model is relevant to human aortic valve stenosis.

**Supplementary Information:**

The online version contains supplementary material available at 10.1186/s13578-023-00980-1.

## Background

As an essential signaling pathway in embryonic development, the hedgehog (Hh) pathway is critical for maintaining tissue polarity and stem cell population. Smoothened (SMO), the seven transmembrane domain-containing protein, serves as the critical signal transducer, whose function is inhibited by another transmembrane protein, Patched (PTC). An active Hh ligand (Shh, Ihh, Dhh) binds to its receptor PTC to release this inhibition, allowing SMO to signal downstream, eventually activating Gli transcription factors. As transcription factors, Gli molecules can associate with specific consensus sequences located in the promoter region of the target genes and regulate target gene expression [1, 2]. The general signaling mechanisms of the Hh pathway are conserved from flies to humans [3].

Hedgehog signaling activation has been observed in several human pathological conditions, including cancer and fibrosis [4–13]. Basal cell carcinomas (BCCs), the most common type of human cancer, are known to be caused by activation of the hedgehog pathway, via loss of function mutations of *PATCHED-1* (*PTCH1*) or gain of function mutations of *SMOOTHENED* (*SMO*). We previously demonstrated that targeted expression of active *SMO*, *SMOM2*, resulted in phenotypes resembling human BCCs [14]. Small molecule compounds have been developed to treat BCCs, and two such molecules that target SMO have been approved by FDA to treat locally advanced and metastatic BCCs [15]. In addition, mutations of *PTCH1* and *SMO* are found in other types of cancer, where hedgehog signaling is activated in the epithelial cells (cancer cells) [16].

Activation of hedgehog signaling is also observed in cancer stromas or non-cancerous pathological conditions, such as fibrotic tissues [6–12]. However, it is not known whether hedgehog signaling is an outcome of fibrosis or a major driver for fibrosis. We hypothesize that activation of hedgehog signaling is sufficient to drive fibrosis in mouse models. To test our hypothesis, we generated mice with active SmoM2 expression under the control of fibroblast-specific promoter-1 (FSP1), and examined the phenotypes in mice. The relevance of the mouse model to the human conditions was assessed using human specimens with fibrosis (aortic valve fibrosis) by examining the expression of a hedgehog target gene, *GLI1*. These data will be presented in the results, and the clinical implications will be discussed.

## Results

### Phenotypes of FSP1-Cre/SmoM2 mice

**Fig. 1:**
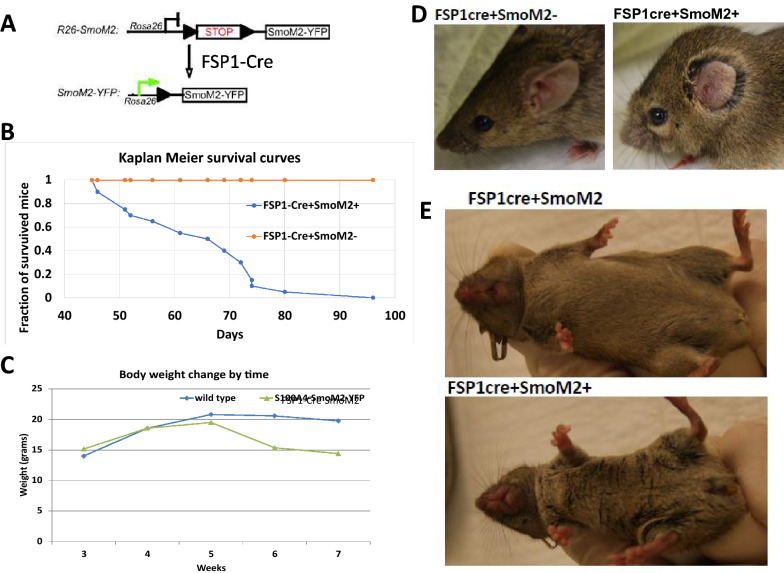
Physical features of FSP1-cre + /SmoM2 + mice. **A** shows a diagram for mouse mating. **B** shows Kaplan meier survival curves from the control and FSP1-cre + /SmoM2 + mice (p = 0.0005). Mice with SmoM2 expression exhibit progressive weight loss (**C**), ear swelling (**D**)**,** and coarse fur (**E**) compared to control mice

In comparison with the control (FSP1-Cre^−^/SmoM2^+^; n = 20) mice, all SmoM2 expressing (FSP1-cre^+^/SmoM2^+^, n = 50) mice died within 4 months. Kaplan -Meier survival curves showed poor survival of FSP1-cre^+^/SmoM2^+^ (see Fig. 1B), p = 0.0005). We observed that FSP1-Cre^+^/SmoM2^+^ mice started to lose their body weights from week 6–7 (Fig. 1C), whereas the control FSP1-cre^+^/SmoM2^−^ mice maintained their normal gain of weights following growth. Closer examination of physical features showed that FSP1-cre^+^/SmoM2^+^ mice had swollen ears and facial skin (Fig. 1D) with coarse fur (Fig. 1E). Alopecia was observed in some FSP1-cre^+^/SmoM2^+^ mice (*n* = 15 out of 50 FSP1-cre^+^/SmoM2^+^mice). Interestingly, we also observed a smaller thymus in FSP1-cre^+^/SmoM2^+^ mice (Additional file 1: Figure S1). These results indicate that FSP1-cre driven expression of SmoM2 is the major factor leading to mouse mortality.

**Fig. 2:**
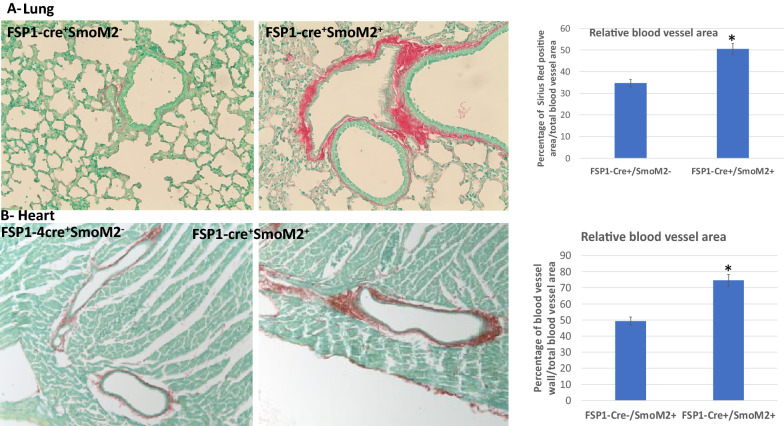
Fibrotic phenotypes in FSP1-cre/SmoM2 mice. **A** shows detection of fibrosis by Sirius Red staining (shown in red) in vasculature of lung, while **B** shows fibrosis (shown in red) in heart vasculature. The relative level of vasculature fibrosis was indicated by the percentage of Sirius Red positive area in the total blool vessel area, and the average from 10 blood vessels was shown at the right of each figure. * indicates p < 0.05

We first examined FSP1 promoter activity in different organs by crossing FSP1-cre transgenic mice with mTmG reporter mice [17]. In previous studies, FSP1 promoter activity is shown in skin [18] and blood vessel (pericytes and endothelial cells) [19] in mice. We were particularly interested in the phenotypes of organs and tissues in FSP1-cre^+^/SmoM2^+^ mice, including the heart, lung and thymus. As shown in Additional file 1: Figure S2, FSP1 promoter activity was observed in mouse aortic valves (shown as GFP expression in the tissue sections indicated by arrows at the top of Additional file 1: Figure S2A). We also detected FSP1 promoter activity in the epithelial cells of thymus (Additional file 1: Figure S1A). Previous work shows that keratinocyte-specific expression of SmoM2 induces BCC-like tumors [20]. In addition, thymus-specific hedgehog signaling is required for the maintenance of low differentiation status of T cells [21], thus SmoM2 expression is anticipated to cause reduced expansion of T cells as shown in our supplemental data (Additional file 1: Figure S1). Tissue sections revealed widespread fibrosis in the vasculature system of lung and heart (Fig. 2A–B).

### Heart abnormality in FSP1-cre^+^/SmoM2^+^ mice

**Fig. 3:**
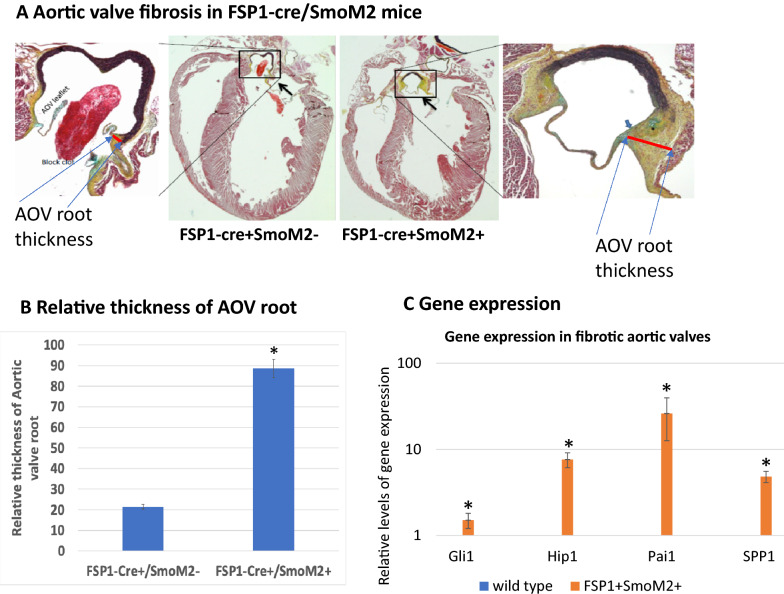
Aortic valve fibrosis in FSP1-cre^+^/SmoM2.^+^ mice. **A** shows pentachrome staining of fibrotic tissue (yellow suggestive of collagen diposition). Please note collagen deposition and size of the aortic valve hinge region and annulus. * shows the site with potential calcification. **B** shows the average of the aortic valve root thickness from 8 mice. # indicates significant difference (p < 0.05) between FSP1-cre + Smo- (or wild-type) mice and FSP1-cre + Smo + mice. **C** shows gene expression levels of hedgehog target genes Gli1, Hip1, and other genes (Pai1 and Spp1) known to be involved in fibrosis. # indicates significant changes in comparison with aortic valves from control mice (p < 0.05). The control value is set as 1

Heart abnormality was observed in 7 week-old FSP1-cre^+^/SmoM2^+^ mice. The heart body weight ratio in the FSP1-cre^+^/SmoM2^+^ mice was slightly higher but insignificant in comparison with the control mice (FSP1-cre^+^/SmoM2^−^, Additional file 1: Figure S3, p = 0.3). This abnormality was not observed in fetal or young FSP1-cre^+^/SmoM2^+^ mice (< 4 weeks), excluding the possibility that expression of SmoM2 results in abnormal cardiac morphogenesis. We found that all FSP1-cre^+^/SmoM2^+^ mice at week 7 or older exhibited fibrosis (yellow stain in Fig. 3A) in aortic valves, with calcification in some areas of aortic valves (indicate by * in Fig. 3A). FSP1 promoter activity was detected in mouse aortic valves (Additional file 1: Figure S1). Consistent with the genotype, high expression of Hh target genes Gli1 and Hip1 in the fibrotic tissues (Fig. 3B). We also observed elevated expression of Pai1 and Spp1, two known genes often detected in fibrotic tissues (Fig. 3B).

**Fig. 4:**
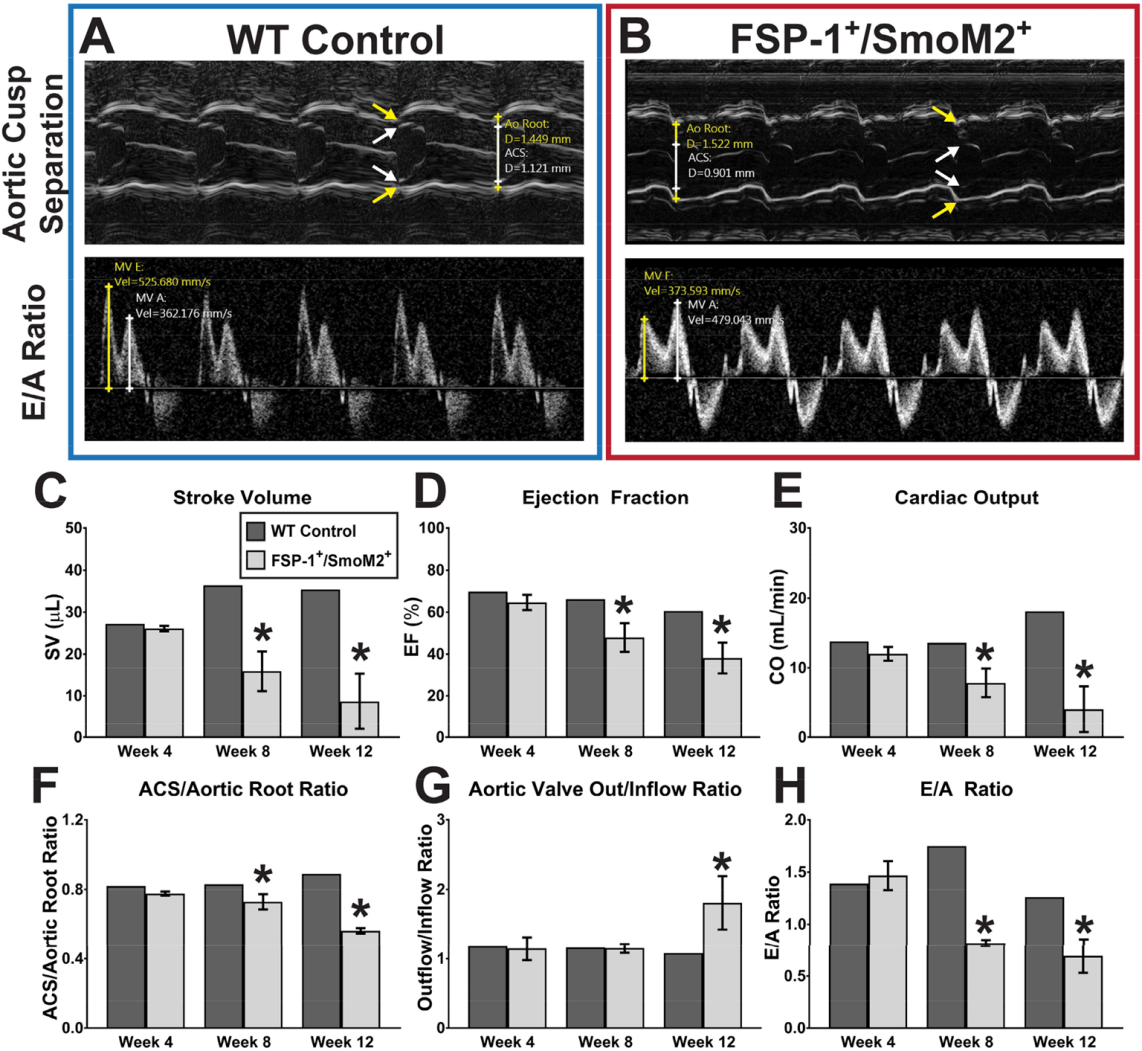
Echocardiographic analyses of aortic valve and heart function. Representative M-mode and PW Doppler images of the aortic valve (top) and transmitral flow (bottom) in both **(A)** WT-control and **(B)** FSP1^+^/SmoM2^+^ mice are shown. Significant systolic dysfunction is observed in FSP1^+^/SmoM2^+^ mice, as shown by a progressive decline in **(C)** stroke volume, **D** ejection fraction, and **E** cardiac output starting at week 8. **F** Aortic cusp separation, normalized to aortic root diameter, progressively decreased starting week 8 in FSP1^+^/SmoM2^+^, suggesting a stiffer aortic valve. **G** An increase in aortic valve outflow velocity, relative to inflow velocity, is consistent with aortic stenosis. **H** FSP1^+^/SmoM2^+^ mice exhibited E/A ratio inversion, suggesting impaired diastolic relaxation of the left ventricle

Aortic valve stenosis is major phenotype of aortic valve diseases, and we assessed aortic valve function using echocardiography. We measured aortic cusp separation (ACS) and the ratio of the early (E) to late (A) ventricular filling velocities in control (shown in Fig. 4A) and FSP1-cre^+^/SmoM2^+^ (shown in Fig. 4B) mice. Cardiac systolic function is reduced in 8 and 12- wk FSP1-cre^+^/SmoM2^+^ mice, as indicated by reduced stroke volume (Fig. 4C), ejection fraction (Fig. 4D), and cardiac output (Fig. 4E). As shown in Fig. 4F, aortic cusp separation (ACS)/aortic root ratio decreased in 8 and 12-week FSP1-cre^+^/SmoM2^+^ mice, suggestive of elevated stiffness of aortic valves following SmoM2 expression. We belived that aortic valve stiffness is a result of fibrosis or calcification in the affected leaflets. We also observed an increase in aortic valve outflow/in flow ratio in 12-wk FSP1-cre^+^/SmoM2^+^ mice (Fig. 4G), indicating aortic valve stenosis. Furthermore, FSP1-cre^+^/SmoM2^+^ mice also exhibited a decrease in cardiac diastolic function, as suggested from reduced E/A ratio (Fig. 4H). Taken together, these results indicate that FSP1-cre^+^/SmoM2^+^ mice desplayed fibrosis in aortic valves, and exhibited aortic valve stenosis.

### Elevated expression of hedgehog target gene GLI1 in diseased human aortic valves

**Fig. 5:**
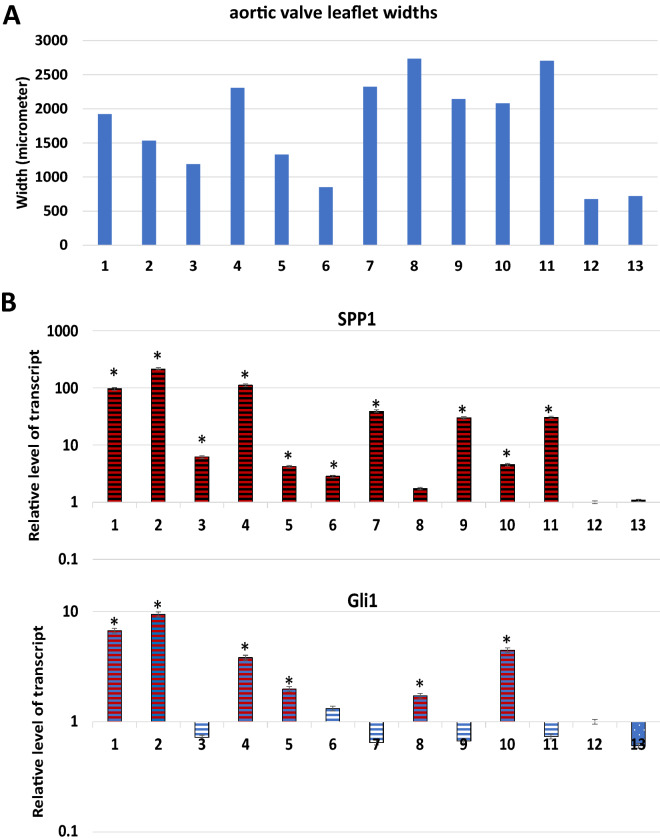
Analyses of normal and diseased aortic valve leaflets. **A** shows the width of aortic valves; **B** shows gene expression in human aortic valves, specimen# 12 and #13 were normal aortic valves. * indicates significant changes (in comparison with normal valves) (p < 0.05)

We sought to correlate our mouse model to human aortic valve diseases. Explanted human aortic valves were collected from patients with AVD or normal controls. As shown in Fig. 5A, we observed histologically that the width of diseased aortic valves were twice that of normal aortic valves (specimen #12 and #13 are the normal aortic valves, see Table 1 for specimen information). Furthermore, we measured gene expression of the hedgehog pathway in human aortic valves, and found that diseased aortic valves had elevated expression of the hedgehog target gene GLI1 in 6 out 11 cases, whereas expression of SPP1 was up in 10 out of 11 cases (Fig. 5B). These results indicate that the hedgehog signaling pathway is activated in over half of the diseased aortic valves with stenosis or regurgitation.

**Table 1:** A list of the specimens used in this study

Patient number	
1	Bicuspid aortic valve, moderate AS and severe AR
2	Unicuspid aortic valve, moderate to severe AR, mild AS
3	Bicuspid aortic valve, severe AS, severely calcified
4	Bicuspid aortic valve, moderate to severe AR
5	Tricuspid aortic valve, severe AR
6	Tricuspid aortic valve, severe AR, possible Marfan
7	Bicuspid aortic valve, severe AS, severe calcification
8	Bicuspid aortic valve, moderately calcified, moderate to severe AR and AS
9	Bicuspid/unicuspid aortic valve with mild AS and moderate AR; severely calcified
10	Bicuspid/unicuspid aortic valve with mild AS and moderateAR; severely calcified
11	Bicuspid aortic valve, severe AS, severely calcified
12	Non-ischemic dilated cardiomyopathy, normal aortic valve
13	Non-ischemic dilated cardiomyopathy, normal aortic valve

## Discussion

Our study provides direct evidence that activating hedgehog signaling can accelerate fibrosis development. Due to the short-life span of the FSP1-cre^+^/SmoM2^+^ mice (less than 4 months), we only observed fibrosis in vasculature in lung and heart (Fig. 2) and aortic valves (Fig. 3). If an inducible expression of cre recombinant is used, we anticipate more wide-spread fibrosis in SmoM2 expressing tissues.

Because FSP1 promoter-driven expression of cre is not limited to fibroblasts, there are several phenotypes not directly associated with fibrosis in FSP1-cre^+^/SmoM2^+^ mice. For example, 7-week old FSP1-cre^+^/SmoM2^+^ mice had very small thymus (Additional file 1: Figure S1). No such differences between FSP1^+^/SmoM2^+^ mice and the control mice were observed in 4 week old mice. Further analyses indicated that T cell differentiation into double positive cells in thymus was blocked in 7 week old FSP1-cre^+^/SmoM2^+^ mice but was normal in 4 week old mice (Additional file 1: Figure S1C). These results are consistent with previous studies on hedgehog signaling in T cell differentiation [22]. The exact mechanism of reduced size in thymus by SmoM2 expression is currently under investigation.

Aortic valve stenosis is major phenotype of aortic valve diseases. In the US, approximately 25% of the population over 65 develops aortic valve disease (AVD) [23], and many require aortic valve replacement (AVR) [24]. At present, there are no medical interventions capable of delaying or halting AVD progression. Echocardiography, particularly M-mode and pulsed-wave (PW) Doppler imaging, can quantitatively assess aortic valve function in mice and humans [25]. We believe that our studies may provide foundation for novel therapeutics for AVD. However, not all diseased aortic valve cases had elevated expression of the hedgehog target gene GLI, indicating that regulation of the hedgehog pathway is not homogenous in all diseased aortic valves. Our data did not find direct association of GLI expression with disease progression or the severity of calcified valves. One explanation could be that aortic valve abnormality may not be all the same at the molecular level. Further gene expression analyses of diseased aortic valves will be needed to identify the gene signature that is associated with high GLI expression. Second, genes other than GLI may mediate Hh signaling [26–28].

In summary, we demonstrate that activating the hedgehog signaling pathway is sufficient to drive fibrosis in mice. We showed that the FSP1-cre^+^/SmoM2^+^ mouse model is relevant to human pathological conditions for aortic valve stenosis, as we found that 6 out of 11 stenosed human aortic valves exhibit increased expression of hedgehog target gene GLI.

## Materials and methods

### Use of explanted human aortic valves

The use of explanted human aortic valves was approved by the Institutional Research Board of Indiana University School of Medicine (IRB protocol 1509977311).

### Animal use

All animal studies were approved by the Institutional Animal Care and Use Committee of Indiana University (IACUC # 20122). Male and female mice were fed with normal chow. Mice with skin lesion or loss of weight were monitored daily, with body weight measured weekly. FSP1-cre (Jackson Laboratory stock#000664), R26SmoM2 (shown as SmoM2 in the text, Jackson Laboratory stock#005130) and mTmG (Jackson Laboratory stock#007576) were purchased from The Jackson Laboratory, and breeding was set up according to the needs of the study (see details in results).

### Echocardiography of aortic valves and cardiac function

We collected all echocardiography images with a Vevo 2100 small-animal ultrasound system (FUJIFILM, VisualSonics Inc., Toronto, Canada) and a 40 MHz linear array transducer (MS550D). Mice were anesthetized with 1–3% isoflurane delivered in 100% O_2_ using a low-flow anesthetic vaporizer (SomnoSuite, Kent Scientific, Torrington, CT, USA). Depilatory cream was applied to remove hair from the left ventral thorax, and the animals were secured on a heated imaging stage. We acquired 2D long- and short-axis images of the left ventricle (LV), and used the Simpson method to approximate end-diastolic and peak-systolic LV volumes, stroke volume (SV), ejection fraction (EF), and cardiac output (CO) following guidelines set by the American Society of Echocardiography [29]. We measured aortic cusp separation (ACS; white arrows in Fig. 4A–B) from M-mode images of the aortic valve, as previously reported by Chu et al. [30], and normalized this value with aortic root diameter (yellow arrows in Fig. 4A–B) to account for differences in heart sizes between mice. Pulsed-wave Doppler images from the four-chamber view was used to measure transmitral flow velocities and calculate E/A ratio, a metric of diastolic dysfunction.

### Histology, special staining, and GFP expression detection in FSP1-cre^+^/mTmG^+^ mice

*Hematoxylin and eosin (H&E)* staining was used to identify tissue morphological changes. Tissues/organs of sacrificed mice were first fixed in 10% buffered neutral formalin for 24 h before embedded in paraffin. Tissue sections with 5 mM thickness were stained with H&E according to our previously published procedure [31]. We employed several methods to detect tissue fibrosis. *Movat Pentachrome Staining* (cat# ab245884) and Masson's Trichrome Staining (cat# ab150686) were performed using Kits from Abcam according to manufacturer’s instruction. *Sirius Red staining* was performed in 0.1% of Sirius red in Saturated aqueous solution of picric acid for 30 min after Fast green staining for 8 min. *GFP expression* in FSP1-cre^+^/mTmG^+^ mice was detected by fluorescent microscope using frozen tissue sections from FSP1-cre^+^/mTmG^+^ mice. DAPI was used to detect nucleus. Measurement of human aortic valve thickness was done with H&E slides, with 3 measurements per section of one valve, and the average number were shown in Fig. 5A. The thickness of aortic valve roots of the mice was measured by Image J, and the average number from 8 mice was presented. For mice, areas of fibrosis in the blood vessels was shown as the percentage of Sirius red positive area in total blood vessel area, and areas were measured by Image J software after Sirius red staining.

### Flow cytometry analysis

Single cells from thymus were dissociated using Accutase (Gibco, CA, USA), resuspended in 5% fetal bovine serum in PBS, and incubated with CD4 and CD8 antibodies on ice for 30 min. CD4 and CD8 expression was analyzed on a FACSCalibur or FACSCanto II (Beckton Dickinson, Franklin Lakes, NJ, USA) using a FlowJo software.

### RNA isolation and real-time PCR

According to the manufacturer's instructions, total RNA was extracted from cells and tissues using TRIzol reagent (Sigma Chemical, St Louis, MO, USA) [31]. The relative abundance of mRNA was calculated by normalizing to GAPDH mRNA. The values from three independent RT-PCR data were used for statistical analyses. One microgram of RNA was used for reverse transcription into cDNA using the First-Strand Synthesis Kit (Roche, Indianapolis, IN, USA). The cDNAs were then diluted and used for real-time PCR with specific probes using Master Mix (Roche) in an ABI7500 detection system (Applied Biosystems, Foster City, CA, USA). Triplicate CT values were analyzed in Microsoft Excel using the comparative CT(CT) method as described by the manufacturer (Applied Biosystems, Foster City, CA). The amount of target (2CT) was obtained by normalization to an endogenous reference (GAPDH) and relative to a calibrator. The probes for real-time PCR were purchased from Applied Biosystems.

### Statistical analysis

Data are presented as mean ± standard deviation (SD) from at least three independent experiments or samples. Statistical comparisons between two groups were performed using a two-tail unpaired t-test 32. P < 0.05 was indicated as the statistically significant difference.

## Supplementary Information

**Additional file 1: Figure S1.** Analyses of thymus in FSP1-cre/SmoM2 mice. **A** shows lineage tracing of FSP1cre activity in thymus, showing high activity in the epithelial cells of thymus. **B** shows the size of thymus from 7-week-old mice. **C** shows reduced CD4 + CD8 + cell population in SmoM2 positive thymus (4 weeks and 7 weeks respectively). **Figure S2**. Tracing FSP1 promoter activity in FSP1-cre/mTmG mice in the aortic valve. **A** shows FSP1 promoter activity (as shown in green- GFP expression). The top panels show the FSP1 promoter reporter activity as indicated in green (shown by yellow arrows). The bottom panels show DAPI staining of nucleus. **B** shows vimentin expression of cells in a mouse aortic valve. The top picture shows vimentin staining, and the bottom picture shows DAPI staining of nucleus. The white bar represents 100 mm. **Figure S3.** Heart/body weight ratio of mice.

## Data Availability

Not applicable.
