# Supplementary material for: Activation of the Hedgehog signaling pathway leads to fibrosis in aortic valves
Source: Cell Biosci. 2023 Mar 2;13:43. doi: 10.1186/s13578-023-00980-1 (PMC9983197; doi:10.1186/s13578-023-00980-1)
Supplement: Supplementary file 1 — Additional file 1: Figure S1. Analyses of thymus in FSP1-cre/SmoM2 mice. A shows lineage tracing of FSP1cre activity in thymus, showing high activity in the epithelial cells of thymus. B shows the size of thymus from 7-week-old mice. C shows reduced CD4 + CD8 + cell population in SmoM2 positive thymus (4 weeks and 7 weeks respectively). Figure S2. Tracing FSP1 promoter activity in FSP1-cre/mTmG mice in the aortic valve. A shows FSP1 promoter activity (as shown in green- GFP expression). The top panels show the FSP1 promoter reporter activity as indicated in green (shown by yellow arrows). The bottom panels show DAPI staining of nucleus. B shows vimentin expression of cells in a mouse aortic valve. The top picture shows vimentin staining, and the bottom picture shows DAPI staining of nucleus. The white bar represents 100 mm. Figure S3. Heart/body weight ratio of mice. [file 13578_2023_980_MOESM1_ESM.pptx]

## Slide 1
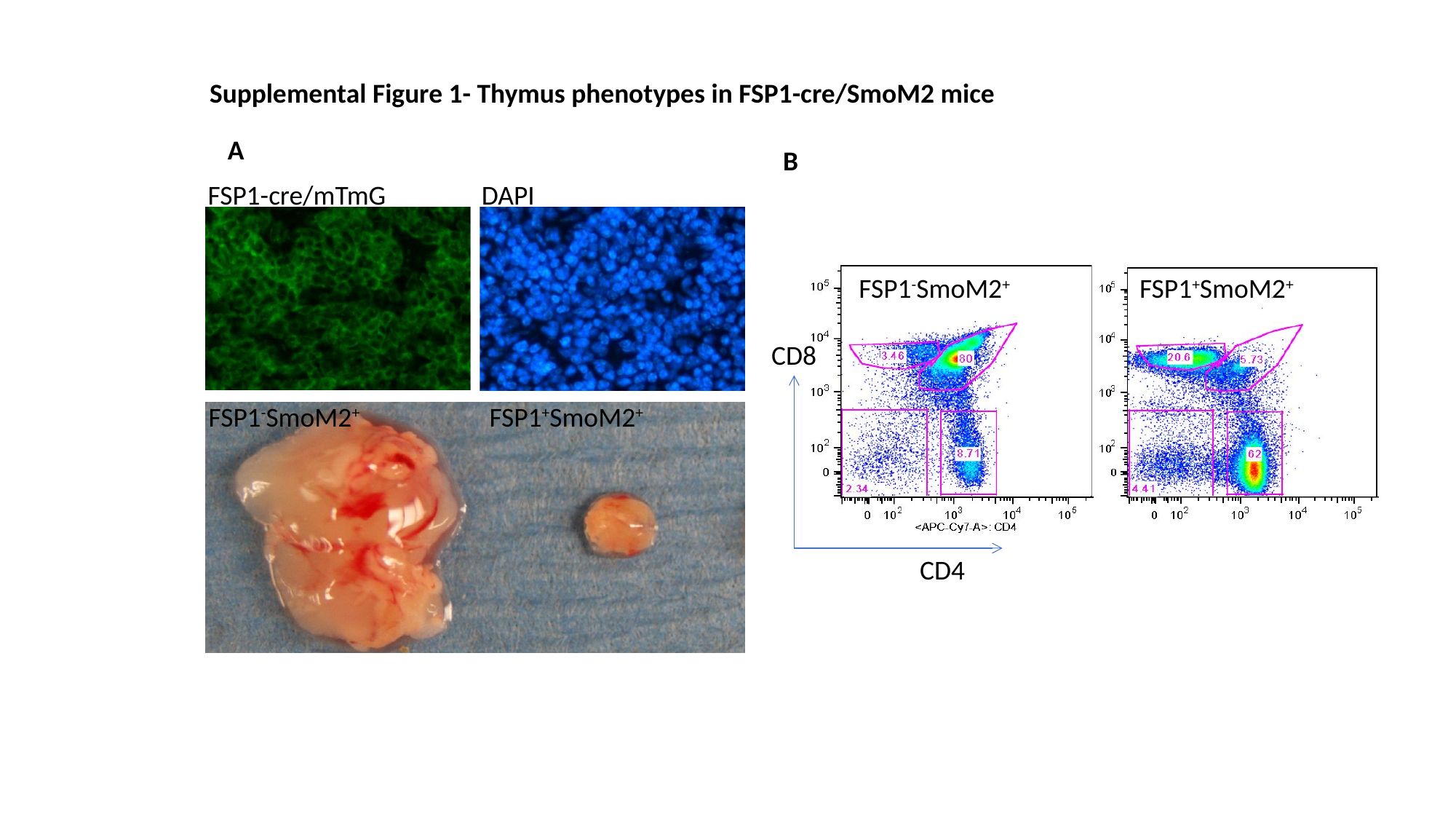

Supplemental Figure 1- Thymus phenotypes in FSP1-cre/SmoM2 mice
FSP1-cre/mTmG
DAPI
FSP1-SmoM2+
FSP1+SmoM2+
CD8
FSP1-SmoM2+
FSP1+SmoM2+
CD4
A
B

## Slide 2
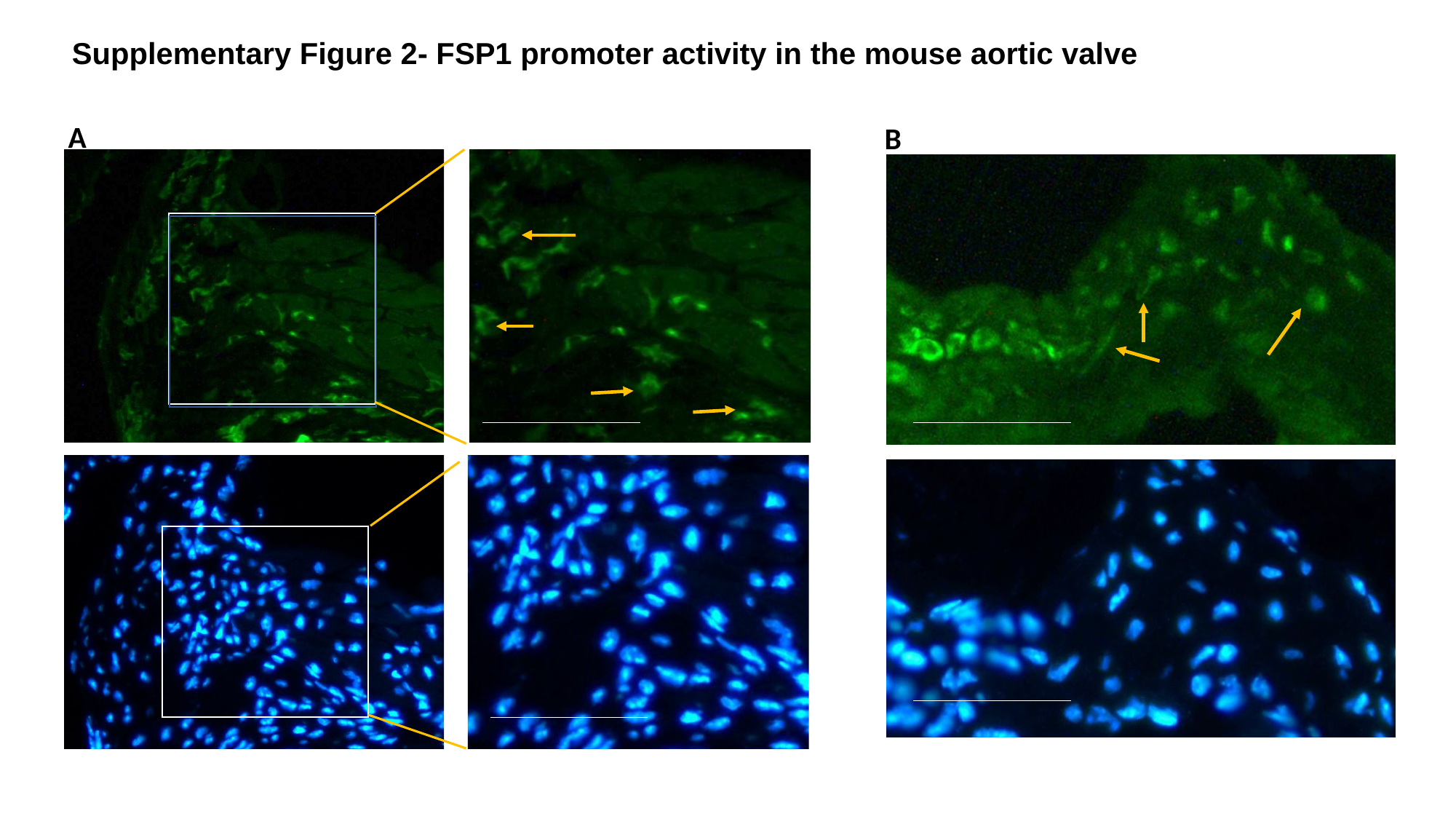

Supplementary Figure 2- FSP1 promoter activity in the mouse aortic valve
A
B

## Slide 3
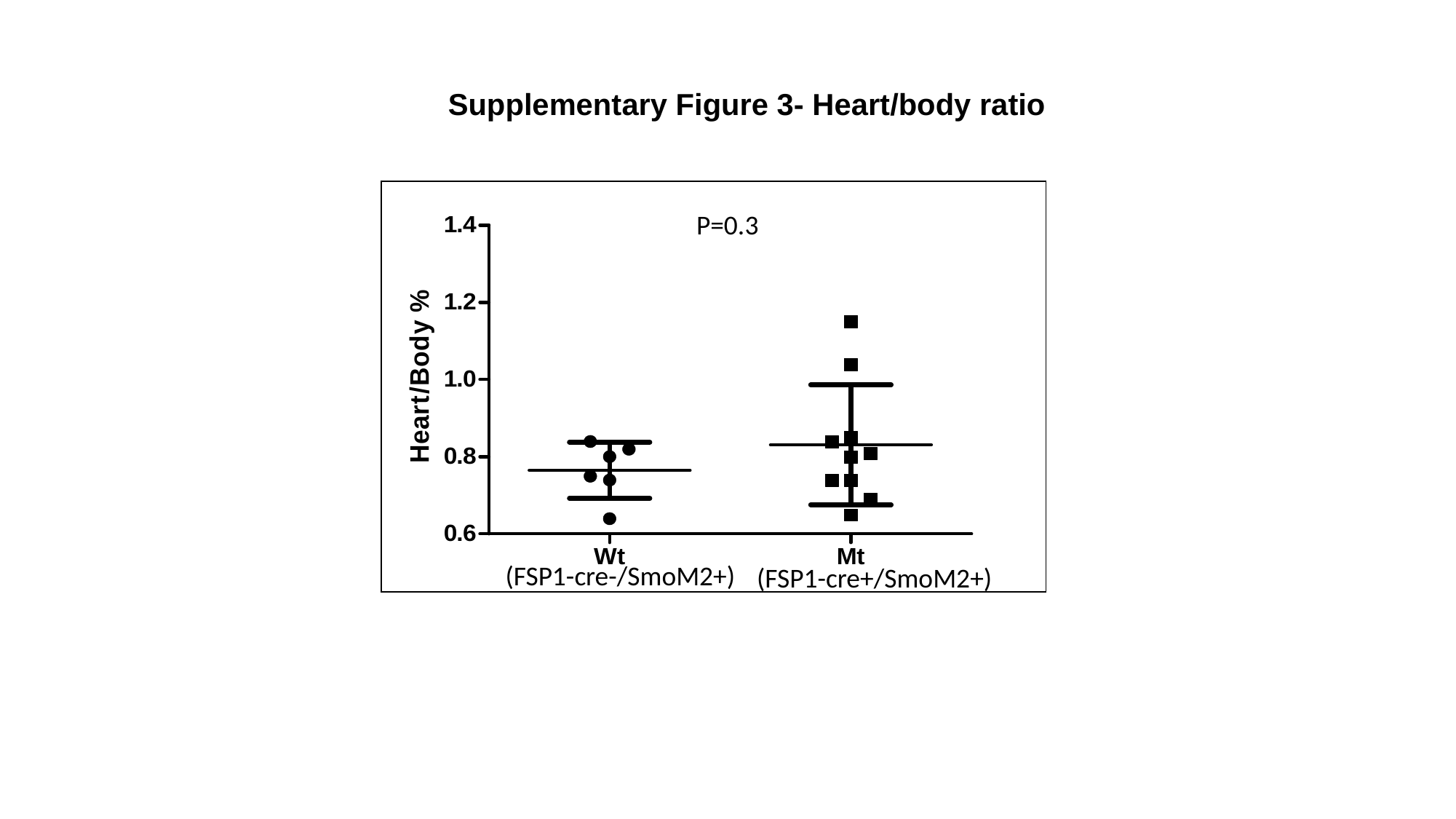

# Supplementary Figure 3- Heart/body ratio
P=0.3
(FSP1-cre-/SmoM2+)
(FSP1-cre+/SmoM2+)
